# Letter on “Pre-hospital transthoracic echocardiography for early identification of non-ST-elevation myocardial infarction in patients with acute coronary syndrome”

**DOI:** 10.1186/s13054-018-2201-z

**Published:** 2018-11-21

**Authors:** Guido Tavazzi, Aleksandar N Neskovic, Bogdan A Popescu, Gabriele Via

**Affiliations:** 1 Department of Clinical, Surgical, Diagnostic and Paediatric Sciences, Anaesthesia, Intensive Care and Pain Therapy Unit, University of Pavia, Pavia, Italy; 2 Emergency Department, Anaesthesia and Intensive Care Unit, Fondazione IRCCS Policlinico S. Matteo, Pavia, Italy; 3 Department of Cardiology, Clinical Hospital Center Zemun, Belgrade, Serbia; 4 Faculty of Medicine, University of Belgrade, Belgrade, Serbia; 5 University of Medicine and Pharmacy “Carol Davila” – Euroecolab Emergency Institute of Cardiovascular Diseases “Prof. Dr. C. C. Iliescu”, Bucharest, Romania; 6 Cardiac Anesthesia and Intensive Care, Cardiocentro Ticino, Lugano, Switzerland

**Keywords:** Focused cardiac ultrasound, Point of care ultrasound, Echocardiography, Myocardial infarction, Non ST-elevation myocardial infarction

We read with interest the manuscript by Bergmann et al. [1] but believe it is fraught by several conceptual and methodological flaws, the main ones being:

- The authors used interchangeably the terms “transthoracic echocardiography”, “focus echocardiography”, and “focus cardiac ultrasound”(FoCUS) without the clear distinction required by the potential major clinical implications [2]; the screening for regional wall motion abnormalities (RWMA) is in fact clearly considered by international consensus beyond the scope of the limited training and application that FoCUS entails [3, 4], and the level of echocardiographic education/competence of emergency physicians was not detailed.
- The authors state: “A diagnosis of NSTEMI was based on the combination of ACS symptoms, lack of ST-segment elevation, and RWMA. Myocardial infarction was excluded in the absence of the latter.” Non-transmural infarctions compromising a small amount of necrotic myocardium may not be detectable on 2D-echo. It has been shown that RWMA detectable by echocardiography occur if resting coronary flow is reduced by > 50%, if > 20% of myocardial thickness is jeopardized by actual ischemia/necrosis, or if at least 1–6% of the left ventricle mass is involved [5].
- Previous myocardial infarction is indicated as an exclusion criterion. But a screening for myocardial scars or signs of pre-existing left ventricle disease was omitted from the exam, and a subsequent re-reading of the images by a blinded expert was omitted too, which may have led to potential false positives in non-ST-segment myocardial infarction (NSTEMI) diagnosis.

It remains obscure how the authors can conclude that “No evidence of myocardial infarction was found in any patient with NSTE-ACS without RWMA in the pre-hospital TTE” and later state that NSTEMI was conclusively diagnosed in two patients without RWMA [1]. We agree, as recommendations do [2], that pre-hospital ultrasound has the potential to lead to earlier diagnosis and faster treatment in acute cardiac patients. But, based on questionable methodology and unclear data, this study conveys the equivocal message that FoCUS has sufficient diagnostic accuracy for NSTEMI.

## Abbreviations

ACS: Acute coronary syndrome
FoCUS: Focus cardiac ultrasound
MI: Myocardial infarction
NSTE-ACS: Non-ST-elevation acute coronary syndrome
NSTEMI: Non-ST-segment myocardial infarction
RWMA: Regional wall motion abnormality
TTE: Transthoracic echocardiography
